# Impact of COVID-19 on the Mental Health of Healthcare Workers: A Cross-Sectional Study From Pakistan

**DOI:** 10.3389/fpubh.2021.603602

**Published:** 2021-04-26

**Authors:** Khezar Hayat, Muhammad Arshed, Iqra Fiaz, Urooj Afreen, Faiz Ullah Khan, Tahir Abbas Khan, Muhtar Kadirhaz, Sundus Shukar, Azwa Saeed, Muhammad Rouf Gill, Yu Fang

**Affiliations:** ^1^Department of Pharmacy Administration and Clinical Pharmacy, School of Pharmacy, Xi'an Jiaotong University, Xi'an, China; ^2^Center for Drug Safety and Policy Research, Xi'an Jiaotong University, Xi'an, China; ^3^Shaanxi Centre for Health Reform and Development Research, Xi'an, China; ^4^Research Institute for Drug Safety and Monitoring, Institute of Pharmaceutical Science and Technology, China's Western Technological Innovation Harbor, Xi'an, China; ^5^Institute of Pharmaceutical Sciences, University of Veterinary and Animal Sciences, Lahore, Pakistan; ^6^Department of Community Health, Faculty of Medicine and Health Science, University of Putra Malaysia (UPM), Seri Kembangan, Malaysia; ^7^Department of Pharmacy, Allied Hospital, Faisalabad, Pakistan; ^8^Department of Pharmacy, Nishtar Hospital, Multan, Pakistan; ^9^Department of Gastroenterology, Pakistan Institute of Medical Sciences, Islamabad, Pakistan; ^10^Department of Zoology, University of Sargodha, Sargodha, Pakistan; ^11^Department of Pharmacy, District Hospital, Toba Tek Singh, Pakistan

**Keywords:** coronavirus, COVID-19, mental health, depression, anxiety, healthcare workers

## Abstract

**Background:** The novel coronavirus (COVID-19) pandemic has significantly increased the rate of mortality and morbidity worldwide due to its rapid transmission rate. The mental health status of individuals could have a negative impact attributed to this global situation. Therefore, this study was intended to explore the symptoms of depression and anxiety among healthcare workers (HCWs) of Pakistan during the ongoing COVID-19 pandemic.

**Methods:** A cross-sectional survey was undertaken by administering a web-based questionnaire between May and June 2020. Two tools, including the Patient Health Questionnaire (PHQ9) and Generalized Anxiety Disorder-7 (GAD-7), were employed to measure anxiety and depression symptoms among HCWs. The data analyses were carried out using descriptive statistics, Man Whitney, and Kruskal Wallis tests.

**Results:** Of 1094 HCWs who participated in this online survey, 742 (67.8%) were physicians, followed by nurses (*n* = 277, 25.3%) and pharmacists (*n* = 75, 6.9%). The survey respondents had a median depression and anxiety score of 5.00 (7.00–3.00) and 8.00 (11.00–5.00), respectively. A considerable number of HCWs (82.2%) utilized online psychological resources to deal with their psychological distress. Female HCWs, nurses, frontline HCWs, and HCWs aged 30–49 years were more likely to suffer from depression and anxiety (*p* < 0.05).

**Conclusion:** During the recent ongoing pandemic of COVID-19, there is a mild level of symptoms of depression and anxiety among HCWs. Our findings call for urgent psychological interventions for vulnerable groups of Pakistani HCWs.

## Introduction

The coronavirus disease 2019 (COVID-19) pandemic is currently a major challenge for public health globally (1). Patients suffering from COVID-19 infection suffer various symptoms, including fever, dry cough, tiredness, loss of smell and taste, shortness of breath, and ultimately death. By 28 January 2021, there were 101,548,107 confirmed COVID-19 cases across the world and 2,187,034 deaths (2). The healthcare system of every country has been paralyzed due to the COVID-19 outbreak, which amplifies the rate of mortality and morbidity. The catastrophic effects of COVID-19 are nearly similar in developing and developed nations; however, the situation could be deadly for countries with poor healthcare systems (3).

Pakistan being a developing country, has 197 million inhabitants. It consists of four provinces Punjab, Sindh, Balochistan, and Khyber Pakhtunkhwa (KPK) and three territories (Gilgit–Baltistan, Islamabad, Azad Jammu and Kashmir) (4). In Pakistan, the first two confirmed cases of COVID-19 were reported on 26 February 2020, which later on challenged the healthcare system of every region of Pakistan (5). Currently, there are 630,471 confirmed cases of COVID-19 and 13,863 deaths in Pakistan. Two provinces, namely, Sindh and Punjab, were hit severely with a significant number of fatalities and COVID-19 cases compared to other regions (Table 1) (6). Numerous unprecedented measures were taken by the government, including travel restrictions, the closure of educational institutes, social distancing, a ban on public transportation, the establishment of dedicated COVID-19 wards, and hospitals to win the battle against COVID-19 (3).

**Table 1:** Statistics of coronavirus (COVID-19) in Pakistan (as of 22 March 2021) (6).

**Province/territory**	**Total cases**	**Active cases**	**Total deaths**	**Total recoveries**
Sindh	263,290	5,081	4,479	253,730
Punjab	199,040	15,360	5,982	177,698
Khyber Pakhtunkhwa	80,037	5,500	2,215	72,322
Islamabad	52,086	5,905	545	45,636
Balochistan	19,342	198	203	18,941
Azad Jammu and Kashmir	11,704	1,013	336	10,355
Gilgit–Baltistan	4,972	13	103	4,856
Total	630,471	33,070	13,863	583,538

Public health emergencies may threaten the safety, health, and well-being of both individuals and communities, which can turn into a range of emotional responses such as depression or psychological diseases (7–9). In Pakistan, more than 5000 HCWs have been diagnosed with COVID-19, of which 58 died, according to a recent report (10). Numerous studies have confirmed anxiety, distress, sleep problems, and depression due to COVID-19 in HCWs working in different fields and positions (11–14). However, the situation is grim for frontline HCWs due to extensive workload, lack of adequate personal protective equipment (PPEs), physical exertion, and risk of nosocomial infections (14, 15). Their resilience may be further jeopardized by loneliness, lack of social contact, risk of catching the virus, illnesses of friends and relatives, as well as dramatic, disturbing shifts in their working pattern (16–18). A recent systematic review found the prevalence of anxiety and depression among HCWs as low as 24.1 and 12.1% and as high as 67.55 and 55.89%, respectively (15). In Pakistan, the psychological impact of COVID-19 among HCWs have so far remained unknown. The performance of individuals could be significantly influenced by psychological well-being. Therefore, this study was designed to investigate the prevalence of depression and anxiety symptoms among HCWs, including physicians, pharmacists, and nurses, during the COVID-19 outbreak in Pakistan.

## Methodology

### Study Design

This was a cross-sectional web-based study carried out between May and June 2020. The mortality and morbidity rate attributed to COVID-19 was highest in two provinces of Pakistan, including Punjab and Sindh (Table 1). Therefore, these provinces were selected as our study sites; however, participants from other regions were also included. The online dissemination of the survey was preferred as it was challenging to field this study offline due to the COVID-19 outbreak. Additionally, Pakistan's government advised all residents to stay home to prevent and reduce the risk of catching COVID-19.

### Survey Tool

A thorough literature review of the relevant articles for designing the survey instrument was conducted (7, 16, 17, 19, 20). The questionnaire validation (face and content validity) was undertaken by a panel of two professors of Pharmacy background, three hospital pharmacists, two medical doctors, and two nurses. The approved questionnaire had 25 items and three sections. In the first section, the respondents' demographic information was asked, including the type of HCW, gender, age, marital status, education, and living place. The second section had seven questions about the exposure of HCWs toward COVID-19 with “yes” and “no” options. Two tools, including the Generalized Anxiety Disorder (GAD-7) and Patient Health Questionnaire (PHQ-9), were used to determine the mental health status in section three. These tools have often been used and validated in various populations as brief screening measures for depression and anxiety (19, 21, 22). Besides, these tools have also been validated on the Pakistani population (23–25).

The GAD-7, which is used to measure anxiety among the respondents, had seven questions with options ranging from 0 (not at all) to 3 (nearly every day) with an overall score of 0–21. The score of the respondents was classified into four distinct categories, such as none (<4), mild (5–9), moderate (10–14), and severe (>15). Likewise, PHQ-9 was used to measure depression among the survey participants and had 9-items. Here, the score of the participants was again classified into five groups, including minimal (1–4), mild (5–9), moderate (10–14), moderately severe (15–19), and severe (≥20). The cut-off score for GAD-7 and PHQ-9 tools was ≥10.

Three questions were asked in the last section to inquire about whether participants have received any psychological services to cope with their mental health issues. Additionally, one question was asked to report the overall health status of HCWs with options ranging from “very poor” to “very good.”

The study was piloted on a small number of subjects prior to the start of the survey to determine the participants' understanding of the survey items. The internal consistency of the questionnaire was acceptable as the value of Cronbach's alpha was 0.799.

### Sample Size

The WHO's recommendations were followed to determine the sample size for this study (26, 27). A total of 384 respondents were required, calculated using an online sample size calculator (Raosoft), assuming a response distribution of 50%, the confidence of interval 95%, and a margin of error of 5% (28).

### Data Collection

Data collection was carried out using the convenience and snowball sampling technique. Different social networking sites such as WhatsApp, Facebook, LinkedIn have been used to administer this survey. Initially, a group of three pharmacists and two physicians was formed, which helped disseminate the survey. Respondents were invited to share the survey with their fellow HCWs. They had the opportunity to fill in the questionnaires by simply following the link. The study's aims were listed on the first page. Besides, confidentiality information, the right of withdrawal, consent, and voluntary participation were also presented. Informed consent was provided by all participants prior to participation. The eligibility criteria of this study include both frontline and non-frontline HCWs living in Pakistan. Participants did not receive any incentives.

### Data Analysis

Sampling characteristics (frequency and percentages) were evaluated using descriptive statistics. Kolmogorov–Smirnov, and Shapiro–Wilk tests were used to check the normality of the data. The Mann–Whitney and Kruskal—Wallis tests were employed for continuous data. The median scores were calculated for anxiety and depression symptoms, and their association was measured with demographics and exposure toward COVID-19. All analyses were conducted using SPSS (SPSS Inc., version 19, IBM, Chicago, IL, USA). A *p* < 0.05 was set to be statistically significant.

### Ethics Approval

Ethics approval was received from Xi'an Jiaotong University to conduct this study (Ref: Phar-2020-012).

## Results

### Demographic Characteristics

A total of 1,094 HCWs (physicians= 742, 67.8%, nurses= 277, 25.3%) and pharmacists= 75, 6.9%) participated in this survey. Most of the participants were female (*n* = 723, 66.1%); single (*n* = 569, 53.0%); and had 30–49 years of age (*n* = 564, 61.6%). Nearly equal number of participants were from Punjab (*n* = 427, 39.0%) and Sindh province (*n* = 429, 39.2%) as indicated in Table 2.

**Table 2:** Demographic characteristics of participants and knowledge score (*n* = 1,094).

**Variable**	**Frequency (*n*)**	**Percentage (%)**
**Type of professional**		
Physicians^*^	742	67.8
Pharmacists	75	6.9
Nurses	277	25.3
**Gender**		
Male	371	33.9
Female	723	66.1
**Age (years)**		
20–29	414	37.8
30–49	564	51.6
ɥ50	116	10.6
**Marital status**		
Single	569	52.0
Married	488	44.6
Others	37	3.4
**Education**		
Graduation	796	72.8
Post-graduation	298	27.2
**Residence**		
Punjab	427	39.0
Sindh	429	39.2
Others^∧^	238	21.8

* *Physicians include general practitioners and specialists*.

∧ *Others include Balochistan, and three territories, including Islamabad Capital Territory, Gilgit-Baltistan, Azad Jammu, and Kashmir*.

### Exposure of HCWs Toward Coronavirus (COVID-19)

A large number of participants, 991 (90.6), reported that they have not been diagnosed with COVID-19; however, 894 (81.7%) said that their friend or neighbor is suffering from COVID-19. Eight hundred and fifty three (78.0%) HCWs were living with a person with suspected COVID-19 symptoms. A vast majority (*n* = 903, 82.5%) of HCWs reported that their current precautionary measures are not helpful enough to prevent the transmission of COVID-19 (Table 3).

**Table 3:** Exposure toward coronavirus (COVID-19) *n* (%).

**Question**	**Yes *n* (%)**	**No *n* (%)**
Have you been diagnosed with COVID-19?	103 (9.4)	991 (90.6)
Do you manage patients diagnosed with COVID-19?	117 (10.7)	977 (89.3)
Has your family been diagnosed with COVID-19?	419 (38.3)	675 (61.7)
Have your friends been diagnosed?	894 (81.7)	200 (18.3)
Have your neighbors (people living in the same community who may or may not know each other) been diagnosed?	894 (81.7)	200 (18.3)
Is there anyone living with you with suspected symptoms?	853 (78.0)	241(22.0)
Are your current precautions adequate to prevent infection?	191 (17.5)	903 (82.5)

The median depression and anxiety scores were significantly higher among those HCWs who were diagnosed with COVID-19 (Median depression score = 2, IQR = 4.00–2.00 vs. Median depression score = 1, IQR=2.00–1.00; *p* < 0.05 and Median anxiety score = 4, IQR = 4.00–2.00 vs. Median anxiety score = 2, IQR = 2.00–2.00; *p* < 0.05) and involved in the COVID-19 management (Median depression score = 2, IQR = 3.00–2.00 vs. Median depression score = 1, IQR = 2.00–1.00; *p* < 0.05 and Median anxiety score = 2, IQR = 4.00–2.00 vs. Median anxiety score = 2, IQR=2.00-2.00; *p* < 0.05) (Table 5).

### Mental Health Status of HCWs

The median depression and anxiety score with IQR of the survey respondents was 5.00 (7.00–3.00) and 8.00 (11.00–5.00), respectively. Most of the respondents had a mild level of depression (*n* = 497, 45.4%), whereas 131 (12.0%) had a moderate to severe level of depression (Table 4). Regarding the severity of anxiety symptoms, 395 (33.3%) had moderate to severe levels of anxiety as their anxiety score was >10.

**Table 4:** Prevalence of depression and anxiety among healthcare workers.

**Mental health status**		**Healthcare workers *n* (%)**
Depression severity	Minimal	466 (42.6)
	Mild	497 (45.4)
	Moderate	70 (6.4)
	Moderately severe	50 (4.6)
	Severe	11 (1.0)
Anxiety severity	Minimal	166 (15.2)
	Mild	563 (51.5)
	Moderate	270 (24.7)
	Severe	95 (8.7)

The median depression score was found to be significantly higher among physicians (Median = 2, IQR = 2.00–1.00; *p* < 0.05) compared to nurses (Median = 1, IQR = 2.00–1.00; *p* < 0.05). Female participants had a higher median depression score than male participants (Median = 2, IQR = 2.00–1.00 vs. Median = 1, IQR = 2.00–1.00; *p* < 0.05). Likewise, participants living in Punjab had a significantly higher median score than those living in other regions of Pakistan (Median = 2, IQR = 2.00–1.00 vs. Median = 1, IQR = 2.00–1.00; *p* < 0.05).

HCWs with higher age (>50 years) had a higher level of depression symptoms than respondents with 20–29 years of age (*p* < 0.05). Similarly, the symptoms of anxiety were noted to be significantly higher among physicians, female respondents, respondents with age >50, and residents of Punjab (Table 5).

**Table 5:** Analysis of depression scores and anxiety scores by sociodemographic characteristics.

**Variables**	**Frequency (*n*)**	**Percentage (%)**	**Depression score**		**Anxiety score**	
			**Median (IQR)**	***p***	**Median (IQR)**	***p***
**Type of professional**						
Physicians^*^	742	67.8	2.00 (2.00–1.00)	0.001	2.00 (2.00–2.00)	<0.001
Pharmacists	75	6.9	1.00 (2.00–1.00)		1.00 (2.00–1.00)	
Nurses	277	25.3	1.00 (2.00–1.00)		2.00 (2.00–2.00)	
**Gender**						
Male	371	33.9	1.00 (2.00–1.00)		2.00 (2.00–0.00)	
Female	723	66.1	2.00 (2.00–1.00)	<0.001	2.00 (2.00–2.00)	0.07
**Age (years)**						
20–29	414	37.8	1.00 (2.00–1.00)		2.00 (2.00–2.00)	
30–49	564	51.6	2.00 (2.00–1.00)		2.00 (2.00–2.00)	
ɥ50	116	10.6	2.00 (2.00–1.00)	0.002	2.00 (2.00–2.00)	<0.001
**Marital status**						
Single	569	52.0	1.00 (2.00–1.00)		2.00 (2.00–2.00)	
Married	488	44.6	2.00 (2.00–1.00)		2.00 (2.00–2.00)	
Others	37	3.4	1.00 (2.00–2.00)	0.01	2.00 (2.00–2.00)	0.58
**Education**						
Graduation	796	72.8	2.00 (2.00–1.00)	0.88	2.00 (2.00–2.00)	0.27
Post-graduation	298	27.2	2.00 (2.00–1.00)		2.00 (2.00–2.00)	
**Residence**						
Punjab	427	39.0	2.00 (2.00–1.00)	0.04	2.00 (2.00–1.00)	<0.001
Sindh	429	39.2	2.00 (2.00–1.00)		2.00 (2.00–2.00)	
Others^∧^	238	21.8	1.00 (2.00–1.00)		2.00 (2.00–2.00)	
**Diagnosed with COVID-19**						
Yes	103	9.4	2.00 (4.00–2.00)	<0.001	4.00 (4.00–2.00)	<0.001
No	991	90.6	1.00 (2.00–1.00)		2.00 (2.00–2.00)	
**Manage patients with COVID-19**						
Yes	117	10.7	2.00 (3.00–2.00)	<0.001	2.00 (4.00–2.00)	<0.001
No	977	89.3	1.00 (2.00–1.00)		2.00 (2.00–2.00)	

* *Physicians include general practitioners and specialists*.

∧ *Others include Balochistan, and three territories, including Islamabad Capital Territory, Gilgit-Baltistan, Azad Jammu, and Kashmir*.

**Figure 1:**
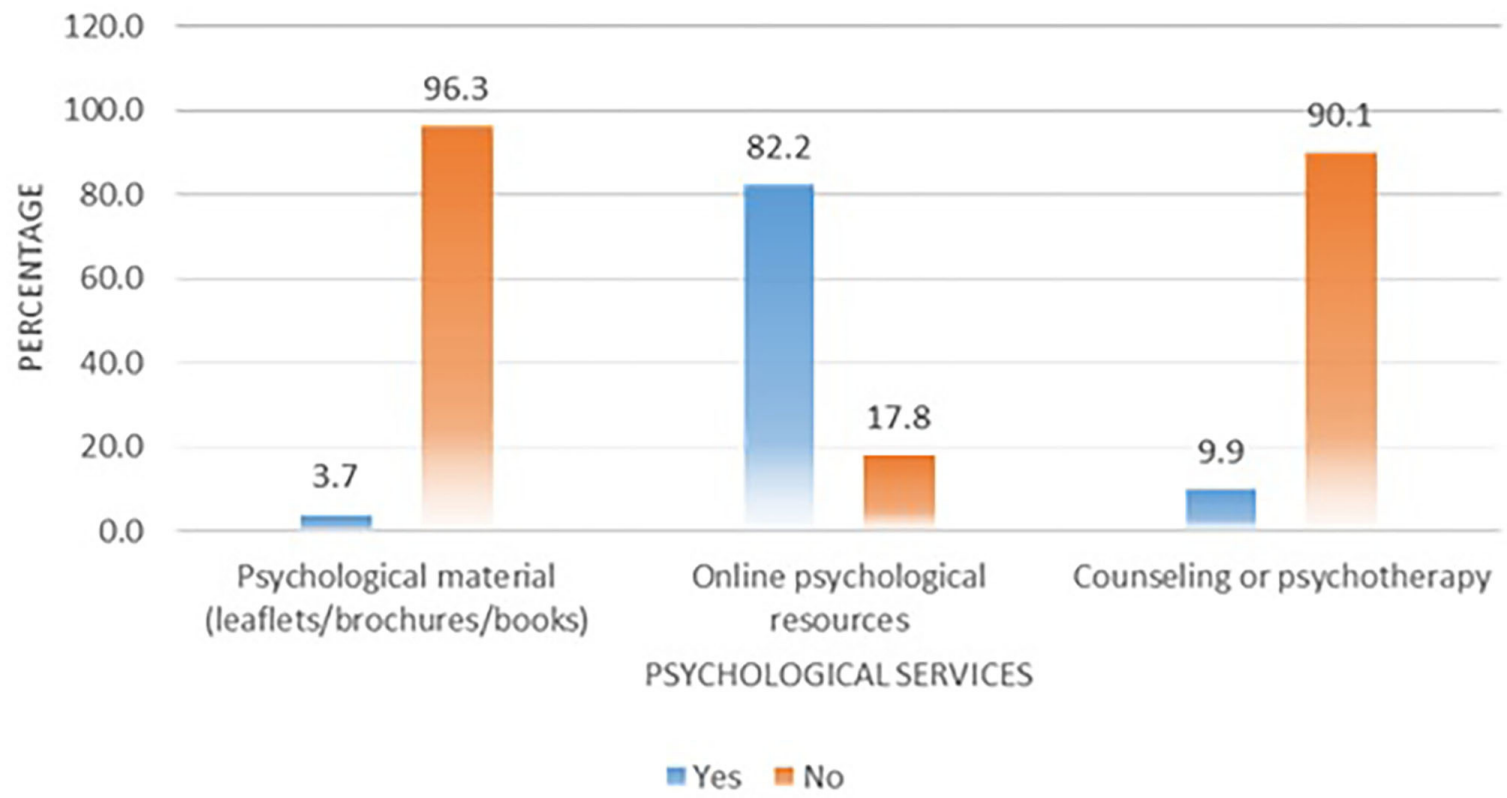
Utilization of various psychological aids by healthcare workers.

Most of the HCWs (82.2%) were using online psychological resources to cope with their mental health issues; however, the use of psychological aids in the form of books/leaflets was uncommon (3.7%). Similarly, only 9.9% of respondents used psychotherapy or counseling to win the battle against psychological problems during the COVID-19 outbreak (Figure 1).

The regression analysis revealed that the depression risk is significantly higher among nurses [Odds ratio (OR) = 3.974, 95% Confidence interval (CI) = 1.829–8.631; <0.001], HCPs dealing COVID-19 patients (OR = 10.820, CI = 6.469–18.099; *p* < 0.001) and HCPs who are diagnosed with COVID-19 (OR = 6.025, CI = 2.985–12.160; *p* < 0.001). Likewise, female HCWs (OR = 0.183, CI = 0.115–0.289; *p* < 0.001), HCWs aged 30–49 years (OR = 0.337, CI = 0.151–0.750, *p* = 0.008), and married HCWs were prone to suffer anxiety (OR = 2.803, CI = 1.051–7.477, *p* = 0.040). The detailed results are presented in Table 6.

**Table 6:** Regression analysis of depression and anxiety among healthcare professionals.

**Variables**	**Frequency (*n*)**	**Percentage (%)**	**Depression**		**Anxiety**	
			**OR 95% CI**	***P***	**OR 95% CI**	***P***
**Type of HCP**						
Physician^*^	742	67.8	Reference			
Pharmacist	75	6.9	1.376 (0.689–2.748)	0.366	6.657 (3.985–11.120)	<0.001
Nurse	277	25.3	3.974 (1.829–8.631)	<0.001	1.958 (0.846–4.530)	0.116
**Gender**						
Male	371	33.9	Reference			
Female	723	66.1	0.766 (0.455–1.288)	0.315	0.183 (0.115–0.289)	<0.001
**Age (years)**						
20–29	414	37.8	Reference			
30–49	564	51.6	1.613 (0.531–4.902)	0.399	0.337 (0.151–0.750)	0.008
>50	116	10.6	1.793 (0.719–4.474)	0.211	0.630 (0.337–1.177)	0.147
**Marital status**						
Single	569	52.0	Reference			
Married	488	44.6	1.331 (0.240–7.370)	0.744	2.803 (1.051–7.477)	0.040
Others	37	3.4	1.250 (0.237–6.597)	0.793	1.745 (0.702–4.337)	0.231
**Education**						
Graduation	796	72.8	Reference			
Post-graduation	298	27.2	1.191 (0.684–2.073)	0.537	1.877 (1.142–3.086)	0.013
**Location**						
Punjab	427	39.0	Reference			
Sindh	429	39.2	1.447 (0.692–3.024)	0.326	3.881 (2.104–7.159)	<0.001
Others^∧^	238	21.8	0.557 (0.271–1.146)	0.112	0.709 (0.418–1.201)	0.200
**Diagnosed with COVID-19**						
Yes	103	9.4	Reference			
No	991	90.6	6.025 (2.985–12.160)	<0.001	45.950 (19.950–105.836)	<0.001
**Manage patients with COVID-19**						
Yes	117	10.7	Reference			
No	977	89.3	10.820 (6.469–18.099)	<0.001	2.249 (1.258–4.020)	0.006

* *Physicians include general practitioners and specialists*.

∧ *Others include Balochistan, and three territories, including Islamabad Capital Territory, Gilgit-Baltistan, Azad Jammu and Kashmir*.

## Discussion

To the best of our knowledge, the current study is the first to outline the mental health status of HCWs in Pakistan. This study found the symptoms of depression and anxiety among a substantial number of survey respondents. Being a physician, female gender, and age of 30–49 years had a significantly higher level of depression and anxiety symptoms.

Like our study, many studies have been conducted in different countries that have detected the presence of depression and anxiety among HCWs (18, 29, 30). Additionally, a recent study undertaken in China found the symptoms of depression and anxiety among a significant number of enrolled HCWs (depression = 50.4%, anxiety = 44.6%), which further affirms our findings (31).

In our study, 9.4% of the HCWs were diagnosed with COVID-19. The risk of catching COVID-19 infection is higher among frontline HCWs than the general community due to their involvement in the cure and treatment of patients with COVID-19 (32). A recent longitudinal study conducted on hospitalized patients with COVID-19 confirmed the presence of hospital-related transmission of novel coronavirus pneumonia, which possibly leads to infecting 40 (29%) HCWs in China (33). In Pakistan, 58 HCWs, including 42 doctors, died due to COVID-19, and more than 5,000 have been tested positive (34). The risk of prevalence of depression and anxiety could surge owing to the increasing rate of COVID-19 infection among HCWs.

A study conducted during the epidemic on 52,730 respondents showed a higher risk of psychological distress among female respondents than males (35). Likewise, in our study, female HCWs had a significantly higher symptoms of anxiety, which is in accordance with the published data during epidemics where a female working in a healthcare system was more prone to develop depression and anxiety (31, 36).

It has been reported previously that the impact of psychological distress varies depending upon gender. Numerous studies have shown that females are 1.6 times more likely to develop mental torment due to the difference in hormonal fluctuations in men and women (37, 38).

Our study shows that respondents with higher age (>50 years) have a significantly higher impact on mental health. The recent literature has highlighted a higher mortality risk (3.6–14.8%) among the elderly population, and this information has been shared on various media platforms. Therefore, older adults are instructed to remain at home and maintain social distancing to cut the risk of COVID-19 infection. However, social isolation is a concern of public health, which could augment the probability of severe mental health issues among older people (39–42). Besides, a case study from India showed that older adults are committing suicide due to a relapse of a depressive illness during the COVID-19 outbreak (43).

A vast majority of the survey respondents (82.5%) of our study reported that their current precautions are inadequate to prevent COVID-19 infection. Globally, since the outbreak of COVID-19, HCWs have faced an unprecedented challenge (44). HCWs accounted for 9.0% and almost 14.0% of confirmed COVID-19 cases in their first month of outbreaks in Italy and Spain, respectively (45, 46). In Pakistan, the HCWs protested because of inadequate personal protective equipment, began a hunger strike, and threatened to halt working (47–49). Owing to this, there is a continuous surge in the number of confirmed COVID-19 cases among HCWs, which in turn will potentiate the risk of mental health issues (10). The WHO has also emphasized the availability of PPE for HCWs to limit their chances of catching the infection (50).

The survey respondents diagnosed with COVID-19 or in contact with COVID-19 patients had significantly higher mental health deterioration than others. Numerous studies have already shown that frontline HCWs and COVID-19 patients are at risk of developing mental disorders owing to extensive workload, inadequate personal protective equipment (PPEs), fear of catching the infection, and lack of social contact (14, 31, 51–53). However, a recent study undertaken in Malaysia reported that both frontline and non-frontline HCWs require similar psychological support (51).

Our study inherits certain limitations. First, this study's generalizability is limited due to its convenience-sampling approach; however, this is an exploratory study that has provided an insight into the current mental health status of HCWs. Second, the study was conducted online by a self-administered questionnaire, which may carry response bias; nevertheless, it was challenging to undertake this study offline because of lockdown in different regions of Pakistan. Third, we have not asked HCPs regarding the nature of their practicing hospital and the mental health status of HCPs working in various health settings such as tertiary care, secondary care and primary care could differ. Regardless of these limitations, this study will stimulate the government to design psychological interventions to minimize mental illness risk among HCWs.

## Conclusion

In conclusion, our study reports a mild level of symptoms of anxiety and depression among most healthcare personals. The risk of mental illness was significantly higher among female HCWs, nurses, frontline HCWs and respondents with age 30–49 years. Follow-up studies with a large sample size are warranted to confirm the results of this study. The onus may be on the government to launch immediate psychological interventions for HCWs of Pakistan.

## Data Availability Statement

The raw data supporting the conclusions of this article will be made available by the authors, without undue reservation.

## Ethics Statement

The studies involving human participants were reviewed and approved by Center for Drug Safety and Policy Research, Xi'an Jiaotong University. The patients/participants provided their online informed consent to participate in this study.

## Author Contributions

KH, IF, TK, and YF: conceptualization. KH, AS, MA, IF, UA, TK, and MG: data curation. KH and FK: formal analysis. YF: funding acquisition, supervision, and writing-review and editing. MA: investigation. KH, UA, TK, MK, and YF: methdology. MA, IF, UA, FK, SS, and MG: project administration. KH: writing-original draft. All authors have read and agreed to the published version of the manuscript.

## Conflict of Interest

The authors declare that the research was conducted in the absence of any commercial or financial relationships that could be construed as a potential conflict of interest.
